# Elucidating the Molecular Origins of the Transference Number in Battery Electrolytes Using Computer Simulations

**DOI:** 10.1021/jacsau.2c00590

**Published:** 2023-02-02

**Authors:** Chao Fang, Aashutosh Mistry, Venkat Srinivasan, Nitash P. Balsara, Rui Wang

**Affiliations:** † Materials Sciences Division, Lawrence Berkeley National Laboratory, Berkeley, California94720, United States; ‡ Department of Chemical and Biomolecular Engineering, University of California, Berkeley, California94720, United States; § Joint Center for Energy Storage Research, Argonne National Laboratory, Lemont, Illinois60439, United States; ∥ Chemical Sciences and Engineering Division, Argonne National Laboratory, Lemont, Illinois60439, United States

**Keywords:** batteries, electrolytes, ion transport, transference number, computer simulation

## Abstract

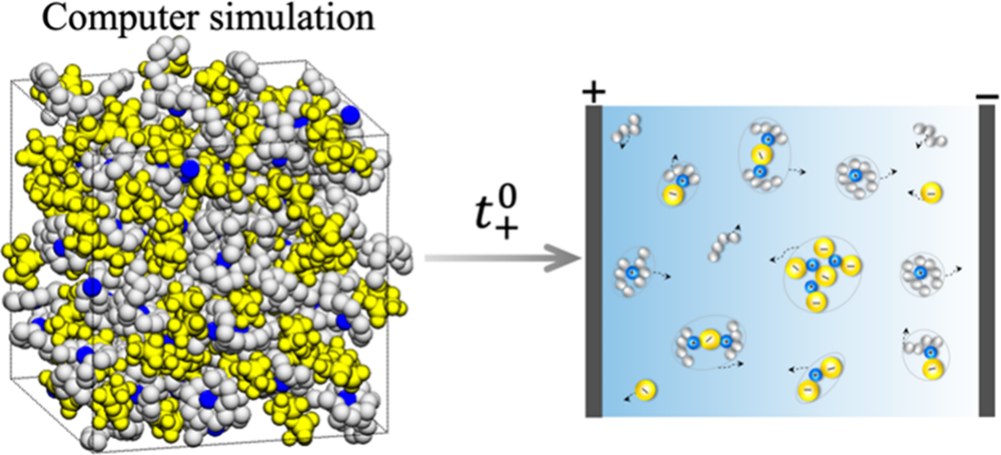

The rate at which rechargeable batteries can be charged and discharged is governed by the selective transport of the working ions through the electrolyte. Conductivity, the parameter commonly used to characterize ion transport in electrolytes, reflects the mobility of both cations and anions. The transference number, a parameter introduced over a century ago, sheds light on the relative rates of cation and anion transport. This parameter is, not surprisingly, affected by cation–cation, anion–anion, and cation–anion correlations. In addition, it is affected by correlations between the ions and neutral solvent molecules. Computer simulations have the potential to provide insights into the nature of these correlations. We review the dominant theoretical approaches used to predict the transference number from simulations by using a model univalent lithium electrolyte. In electrolytes of low concentration, one can obtain a quantitative model by assuming that the solution is made up of discrete ion-containing clusters–neutral ion pairs, negatively and positively charged triplets, neutral quadruplets, and so on. These clusters can be identified in simulations using simple algorithms, provided their lifetimes are sufficiently long. In concentrated electrolytes, more clusters are short-lived and more rigorous approaches that account for all correlations are necessary to quantify transference. Elucidating the molecular origin of the transference number in this limit remains an unmet challenge.

## I Introduction

Electrolytes for rechargeable batteries consist of electrically conducting ions dissolved in solvents. The performance of batteries cells during charging or discharging can only be predicted when continuum properties are known.^1−5^ We base our discussion on Newman’s concentrated solution theory for electrolytes that comprise a binary salt and a solvent. This necessitates the knowledge of three ion transport properties: the conductivity, κ; the salt diffusion coefficient, *D*; and the cation transference number with respect to the solvent velocity, *t*_+_^0^.^3^ While κ and *D* reflect the collective transport of both cations and anions, *t*_+_^0^ reflects the relative transport rate of cations relative to anions. It is defined as the fraction of ionic current carried by cations in an electrolyte of uniform composition.^6,7^ Knowledge of all three transport coefficients (and relevant thermodynamic parameters - the salt activity coefficient and the partial molar volume of the salt) enables modeling the time-dependent relationship between applied current and nonuniform electrolyte composition.^2,8^ The cation current is proportional to the product of cation concentration and velocity; the latter is only defined after one specifies a reference frame. In classic compilations of *t*_+_^0^,^9^ the reference frame used is that of the solvent. Some of the solvent molecules may drift in the presence of ionic current due to transient association with either ion. In sufficiently dilute electrolytes, the fraction of associated solvent molecules will approach zero. Even in this case, the electrolytic phase will have a net velocity as the electrochemical reactions at the electrodes must involve a change in volume.

**Figure 1:**
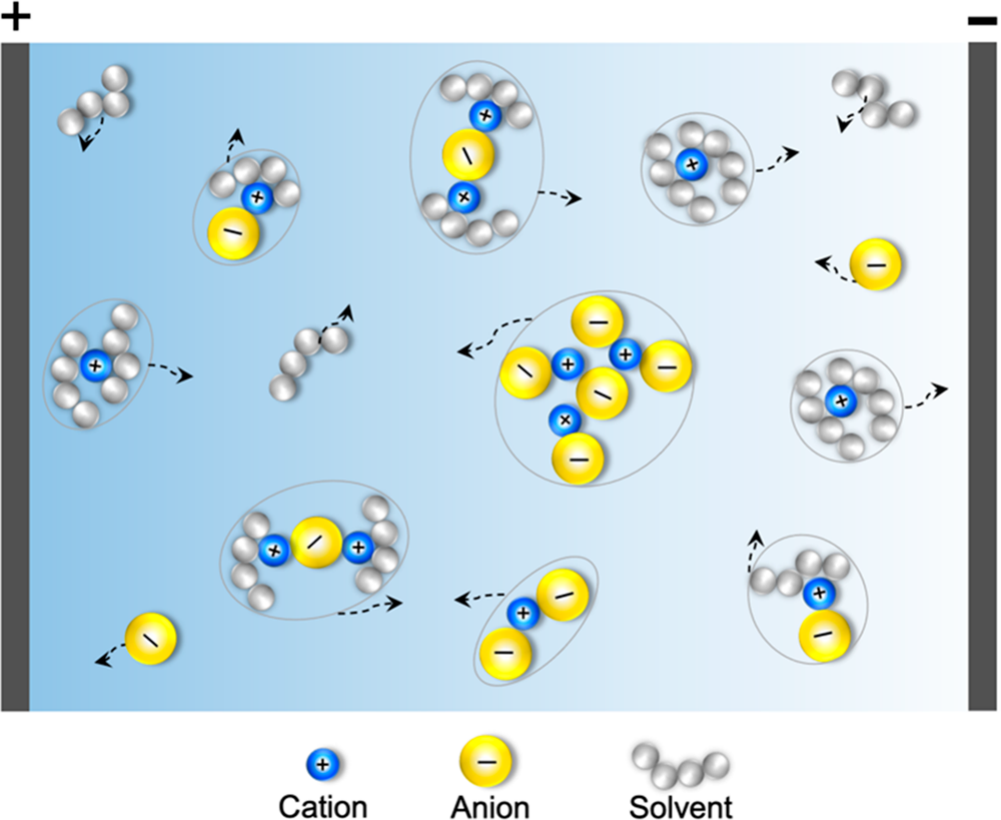
Schematic representation of transient clusters of different types in an electrolyte. Their migration velocities under an applied field are indicated by arrows.

*t*_+_^0^ can be defined in terms of average species velocities that are obtained at the instant the field is applied to an electrolyte of uniform composition^10^
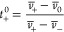
1awhere *v̅*_+_, *v̅*_–_, and *v̅*_0_ are, respectively, the average species velocities of cations, anions, and solvent molecules. It goes without saying that *t*_+_^0^ is independent of the magnitude of the applied field. The velocities are defined as positive if pointing from the positive electrode to the negative electrode. One can, equivalently, define the transference number *t*_+_^M^ with respect to the mass average velocity of the electrolyte (*v̅*_M_):
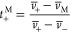
1bThe velocities of individual ions and solvent molecules in different local environments are highly heterogeneous under the electric field. For simplicity, we use *v*_*i*_ (*i* = 0, + , −) to reflect these velocities. While free solvent molecules diffuse randomly (*v*_0_ ∼ 0), cations and solvent molecules in the solvation shell migrate along the electric-field (*v*_+_ > 0, *v*_0_ > 0). Meanwhile, free anions (which generally do not associate with solvent molecules) migrate toward the positively charged electrode (*v*_*–*_ < 0). The scenario becomes much more complicated in the presence of other types of transient clusters, as depicted in Figure 1. The formation of nonmigrating ion pairs and other clusters with no net charge will reduce the magnitude of both *v̅*_+_ and *v̅*_–_. The velocity of charged clusters can be either positive or negative and they can contain varying numbers of solvent molecules, thereby affecting all of the average species velocities.

Unlike κ and *D*, the experimental quantification of *t*_+_^0^ in concentrated solution is typically demanding. Conventional electrochemical characterization of *t*_+_^0^ that is based on concentrated solution theory involves four separate experiments to determine quantities that are, in some cases, indirectly related to the transport parameters.^2,11,12^ This causes large uncertainties.^13^ Electrophoretic-NMR can determine the average velocities of all three species of interest, thereby enabling measurement of *t*_+_^0^ (eq 1a) with greater precision.^10,14−19^ Notwithstanding these challenges, values of *t*_+_^0^ have been reported by numerous researchers.^13,20−32^ A common nontrivial observation centers on negative *t*_+_^0^ in some of electrolytes at high salt concentrations.^11,17,33−36^ It has been widely postulated that negative *t*_+_^0^ originates from the migration of negatively charged ion clusters toward positive electrode as illustrated in Figure 1.^11,33^

Computer simulations, particularly molecular dynamics (MD) simulations, have been extensively used to model ion transport in electrolyte systems.^37−46^ These efforts have the potential to reveal the molecular origins underlying transport bottlenecks; understanding them is crucial for the design and screening of new battery electrolytes. In infinitely dilute electrolytes, ion transport is governed by the Nernst–Einstein equation that assumes uncorrelated motion between ionic species; i.e., ions are fully dissociated and noninteracting. *t*_+_^0^ is only dependent on the diffusive motion of ions, which can be quantified by self-diffusion coefficients via simulation. However, at higher salt concentrations, motions of ions are significantly correlated due to the formation of ion pairs and large ionic aggregates as shown in Figure 1. In addition, correlations between ions are accompanied by the change of ion–solvent interaction. For example, the solvation shell of cations can no longer be entirely made of solvent molecules. The Nernst–Einstein equation is not appropriate in such cases. During the past two decades, various approaches have been proposed to capture ion correlations.^47−52^ While they have been realized through simulations to illustrate the cation transference by different groups, the connection between these approaches as well as their implementation caveats have not yet been scrutinized. In this Perspective, we focus on three approaches proposed for calculating *t*_+_^0^ from MD simulations: two rigorous approaches that describe correlations between species in the system and one approximate approach wherein ionic correlations are simplified to identify discrete clusters (see Figure 1). The three methods are compared by computing *t*_+_^0^ as a function of salt concentration by harvesting the same set of simulations of a model univalent lithium electrolyte. All methods assume the Onsager regression hypothesis, that parameters that govern transport under a finite electric field can be determined by studying the relaxation of concentration fluctuations that occur naturally in a quiescent system that is not perturbed by an external field.

## II Approaches to Quantify *t*_+_^0^

The common step to compute transport properties via equilibrium MD simulations is to first evaluate the time correlation functions from simulation trajectories. In principle, transport properties can be obtained by either differentiating the time correlation functions in the Einstein form with respect to time or integrating them in the Green–Kubo form with respect to time.^53,54^ We use the Einstein form in this perspective. As mentioned above, we discuss three approaches:

(1) From a historical perspective, the first approach for determining the transference number from simulations was proposed by Wheeler and Newman in 2004. This approach builds upon concentrated solution theory.^47,55^
(2) A seemingly independent approach for determining the transference number was proposed by Fong et al. in 2020 based on Onsager transport equations.^52^ This approach has much in common with an approach first proposed by Roling in 2016.^49,56,57^
(3) In the third approach that we discuss, France-Lanord and Grossman proposed in 2019 that estimated self-diffusion coefficients for ionic clusters using the Nernst–Einstein equation could be used to determine the transference number.^51^

**Figure 2:**
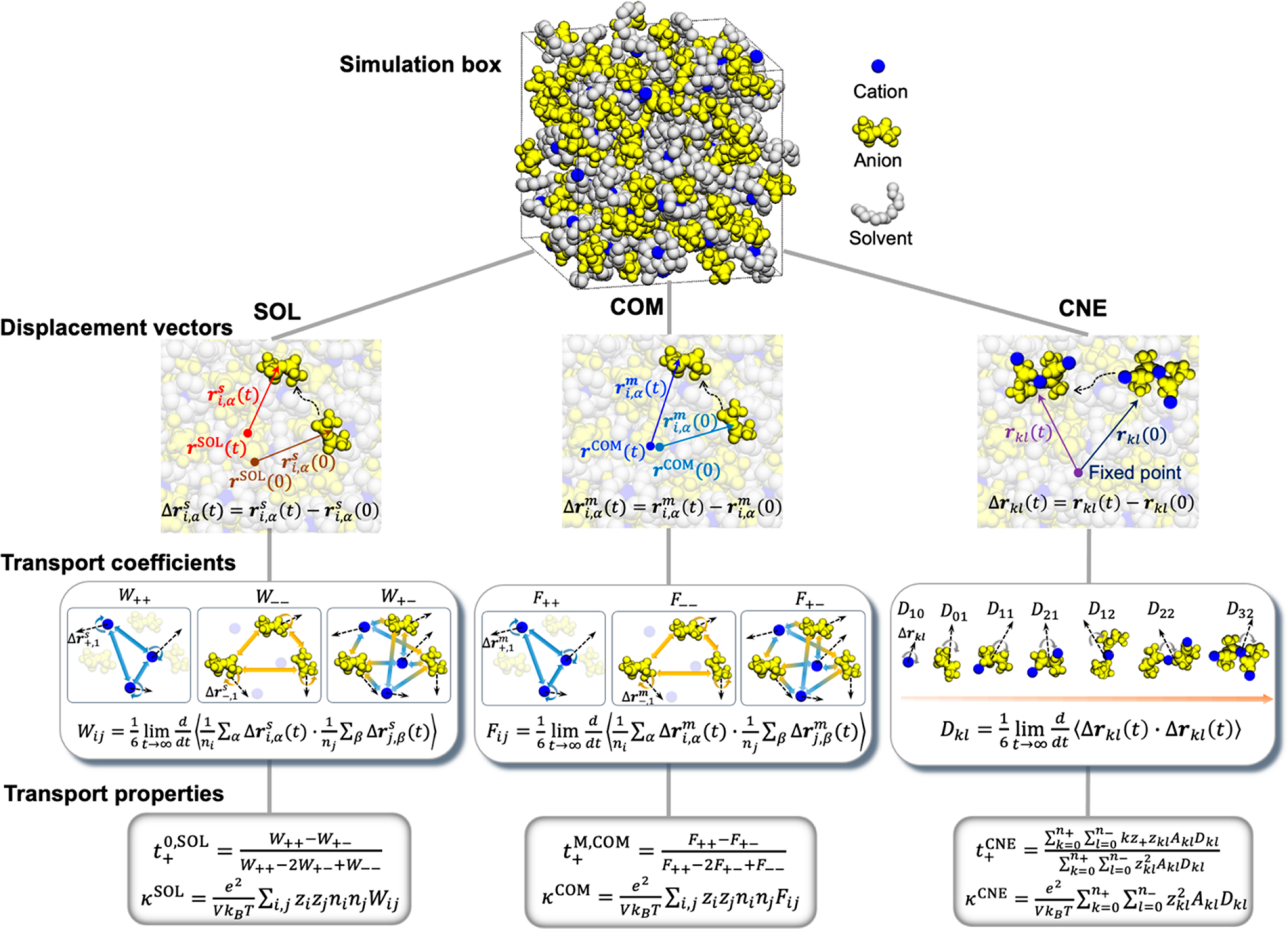
Illustration of the three approaches to calculate transference numbers via equilibrium MD simulations. From 2nd to 4th row: definitions of displacement vectors, species correlations for transport coefficients, and expressions of transport properties.

The three approaches are illustrated in Figure 2 by considering a simulation box consisting of cations, anions, and solvent molecules. The first and second approaches are called “SOL” and “COM”, respectively, to denote the fact that the solvent reference and the center of mass reference were used in the underlying derivations. Both SOL and COM approaches are based on Onsager’s irreversible thermodynamics^4,5^ and are consistent with the concentrated solution theory. The third approach is referred to as “CNE” that corresponds to the cluster Nernst–Einstein.

We calculate conductivity and transference number from the time-dependent displacement of each particle (we use particle to refer to individual molecules and ions). From time 0 to *t*, the displacement vectors for the motion of αth particle of species *i* are defined as
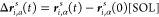
2aand
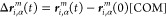
2bThe superscripts *s* and *m* are used to distinguish the reference frames in SOL and COM, which is illustrated in the left two schematics in the second row of Figure 2. *s* represents the particle position with respect to the average position of all solvent molecules in SOL, whereas *m* represents particle position with respect to the center of mass position of the entire system in COM. For CNE, the system is decomposed into different clusters and the displacement vector for an ionic cluster comprising *i* cations and *j* anions is defined as
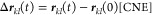
2cThe particle position vectors on the right side of eq 2c are with respect to a fixed point in the simulation box.

The transport coefficients in SOL and COM denote the degree of the dynamic correlations between species. For an electrolyte solution containing *N* species, this corresponds to an *N* × *N* matrix of transport coefficients. Due to the constraint from Gibbs–Duhem relation (or mass balance) and the symmetry of the matrix due to the Onsager reciprocal relation,^47,52,58^ only *N*(*N* – 1)/2 transport coefficients are independent. For a solution comprising three species (cations, anions, and solvent molecules), only three independent transport coefficients are required. In this Perspective, the three transport coefficients quantify cation–cation, anion–anion, and cation–anion correlations. We note in passing that transient clusters shown in Figure 1 are sometimes referred to as “species” in the literature.^59−61^ There is no need to identify clusters in the SOL and COM approaches; the correlation functions will naturally represent their presence. Using the displacement vectors defined above, the transport coefficients for SOL and COM are, respectively, expressed in the Einstein form as
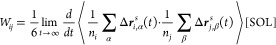
3aand
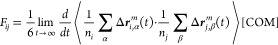
3bwhere *i* and *j* represent species and *n*_*i*_ is the number of particles of species *i*. The expressions of *W*_*ij*_ and *F*_*ij*_ are slightly different from the original transport coefficients reported in refs (47) and (52). They are both written in terms of individual particle displacements for consistency; an equivalent form that is written in terms of collective displacements of species is used in the SOL approach presented in ref (47). *W*_*ij*_ and *F*_*ij*_ have the units of diffusion coefficients.

The dynamic correlations underlying the three independent transport coefficients are illustrated in the third row of Figure 2. For *W*_*ii*_ and *F*_*ii*_, they contain correlations between different ions of the same species as well as the self-correlation of each ion. Specifically, if dot products of each particle are gathered within the ensemble average of *F*_*ii*_ (*a* = β in eq 3b), the self-correlation term is , which is proportional to the self-diffusion coefficient of ion *i* in COM if the motion of center of mass can be neglected.^52,62^ The cross correlation between cations and anions is represented by *W*_+–_ and *F*_+–_. Equations 3a and 3b are similar; however, the dynamic correlations revealed in the SOL and COM approaches are different due to differences in the reference frames. For example, the self-correlation term of *F*_*ii*_ is closely related to the self-diffusion coefficient of ion *i* and is weakly dependent on solvent motion. In contrast, *W*_*ii*_ can be strongly affected by solvent motion.

For CNE, the transport coefficients are the self-diffusion coefficients of all types of charged clusters, including free ions. They are computed from the mean square displacement (MSD) as
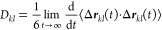
3cwhere *D*_*kl*_ is the self-diffusion coefficient of the cluster comprising *k* cations and *l* anions. The CNE thus approximates correlations in the entire system by assuming that they are dominated by correlations within clusters. Correlations between clusters are ignored. The number of *D*_*kl*_ in CNE depends on the types of charged clusters that appear in the system. In order to calculate *D*_*kl*_, the cluster must not break up for a sufficiently long time. This becomes increasingly problematic as the cluster size increases. Quantification of *D*_*kl*_ is computationally more complex than the calculation of self-diffusion coefficients of species because it involves dynamically tracking the formation and breakup of clusters.

The continuum ion transport properties are given by combinations of the transport coefficients defined in eq 3. We define the computed cation transference numbers for each approach as
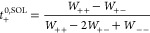
4a
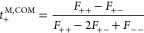
4b
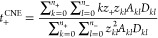
4cwhere *z*_*i*_ is the net charge carried by species *i*. *z*_*kl*_ and *A*_*kl*_ are the net charge and average number of the cluster made of *k* cations and *l* anions, respectively. The Onsager regression hypothesis indicates that *t*_+_^0,SOL^ as defined in eq 4a must be identical to *t*_+_^0^ defined by eq 1a. Similarly, *t*_+_^M,COM^ as defined in eq 4b must be identical to *t*_+_^M^ defined by eq 1b.

The conductivity is given by
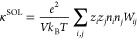
5a
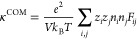
5b
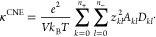
5c*i* and *j* in eqs 5a and 5b denote cation and anion, the total number of which are, respectively, *n*_*i*_ and *n*_*j*_. *e* is the elementary charge, *V* is the system volume, and *k*_*B*_*T* is the thermal energy.

Aside from the above three approaches, we feel it is appropriate to remark on other approaches that have been reported in the literature. Roling et al. derived transport coefficients based on ion displacement relative to a fixed point (which they referred to as the “laboratory reference”). The displacements of solvent molecules are ignored. If the motion of center of mass can be ignored and *v̅*_*M*_ can be approximated as 0, then this approach will lead to results that are similar to the COM approach.^49,56,57^ In addition to approaches based on equilibrium MD simulations, Wheeler and Newman developed a nonequilibrium MD simulation approach that evaluates transport coefficients under an external field.^48^ The transport coefficients approach those obtained by SOL in the limit of zero external field. While we have focused on using displacement fields to calculate the transference number, an equivalent approach based on velocity fields has been used by several researchers.^47,52,63−67^

## III a Case Study in Lithium Electrolyte

The three approaches are compared in a model electrolyte consisting of lithium bis(trifluoromethanesulfonyl)imide (LiTFSI) salt dissolved in tetraglyme (tetraethylene glycol dimethyl ether). The molal salt concentration *m* ranges from 0.2 to 5.5 kg/mol or the ratio *r* between Li^+^ cation and oxygen atom from tetraglyme ranges from 0.01 to 0.24. MD simulations in the *NpT* ensemble (1 bar, 350 K) were performed for LiTFSI/Tetraglyme system using the Gromacs code (version 5.1.4).^68^ Tetraglyme chains are described using the united-atom model that is based on the Transferable Potentials for Phase Equilibria with United Atom description (TraPPE-UA) force field.^69,70^ The compatible all-atom force field is used for Li^+^ and TFSI^–^ ions.^70,71^ The temperature of the system is maintained using the velocity-rescale thermostat^72^ (time constant 1 ps), and the pressure is kept at 1 bar using the Berendsen barostat^73^ (time constant 1 ps). The bonds of the tetraglyme chains are constrained using the LINCS algorithm.^74^ The cutoff scheme (cutoff length 1.2 nm) and the particle mesh Ewald (PME) method^75^ are, respectively, applied to calculate the Lennard-Jones potential and electrostatic interactions. First, the system is packed and energy minimized. Then it is fully relaxed by a set of equilibrium simulations under different temperatures and pressures. Finally, an equilibrium simulation at 1 bar and 350 K is performed up to 1000 ns to obtain trajectories for sampling transport coefficients. At each salt concentration, 4–8 independent simulations are performed to evaluate the error bars of transport coefficients. The trajectories are saved every 10 ps.

**Figure 3:**
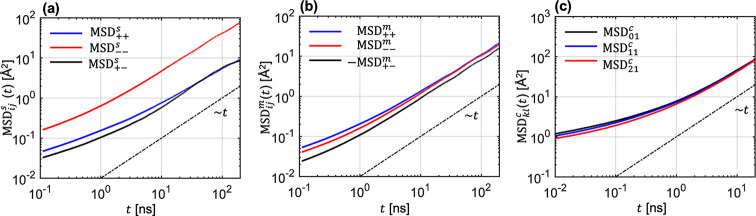
Mean square displacement terms for the three approaches at *r* = 0.16. (a) MSD_*ij*_^*s*^ for *W*_*ij*_. (b) MSD_*ij*_^*m*^ for *F*_*ij*_. (c) MSD_*kl*_^*c*^ for *D*_*kl*_^*c*^. The dash-dotted lines denote the diffusive regime.

For SOL and COM, the transport coefficients are evaluated from the slope of mean square displacement term (MSD_*ij*_) with time, which are respectively defined as
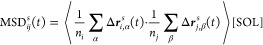
6aand
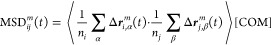
6bMSD_*ij*_s are computed with a window size of 200 ns and the ensemble average in eqs 6a and 6b accounts for all available time origins of each trajectory. Typical MSD_*ij*_s are shown in Figure 3a,b for *W*_*ij*_ and *F*_*ij*_ at a salt concentration of *r* = 0.16, respectively. In the long time limit, the MSD_*ij*_ ∼ *t* scaling is observed. *Fij* and *W*_*ij*_ are fitted over a time interval that is located in this diffusive regime, spanning 1 order of magnitude. Moreover, MSD_+–_^*s*^ and MSD_+–_^*m*^ are of opposite sign at this salt concentration, indicating that cross correlations between cations and anions, i.e., *W*_+–_ and *F*_+–_, are greatly affected by reference frames.

The ion clusters in CNE are identified using the single-linkage algorithm.^51,76^ The linkage between cation and anion is constructed when a Li^+^ is coordinated by a TFSI^–^, i.e., oxygen atoms from TFSI^–^ is within 0.3 nm of the Li^+^. The clusters are reexamined every 10 ps. An individual cluster needs to persist longer than the time window used to successfully compute the right side of eq 3c. The diffusion coefficients of each type of charged clusters are computed from the slope of mean square displacement, which is expressed as^51^
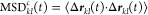
6cTypical MSD_*kl*_^*c*^s are shown in Figure 3c at *r* = 0.16 for small clusters made of 1 anion. They reach the diffusive regime in the later part of the time window. *D*_*kl*_s are obtained by fitting MSD_*kl*_^*c*^s between 10 and 20 ns. Both the time window and fitting regime are similar to those used in the literature.^51^

**Figure 4:**
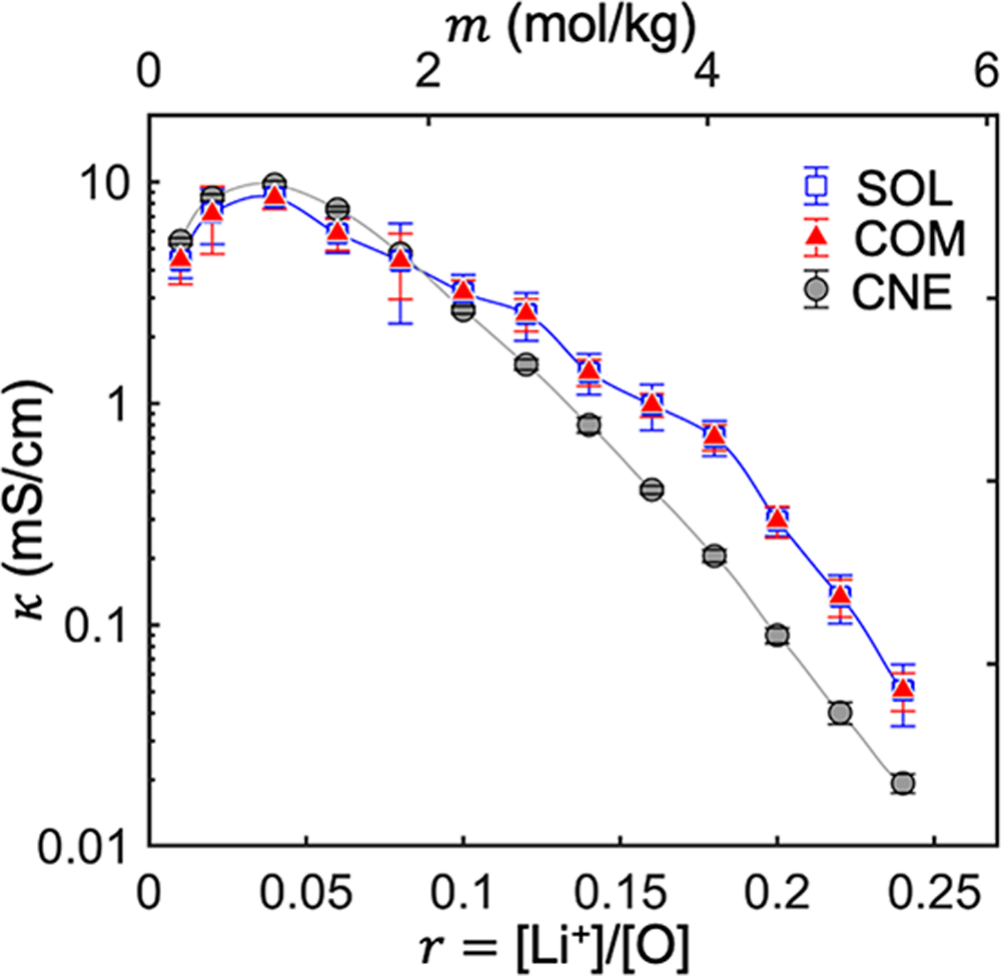
Conductivities from the three approaches as a function of salt concentration *r*.

The conductivities were calculated from the transport coefficients using eq 5, and the results are shown in Figure 4. The salt concentration dependence of κ in LiTFSI/tetraglyme follows the typical behavior observed in many electrolyte systems.^3,77−79^ It increases at low *r* due to the increase of charge carrier concentration. However, friction between species increases with increasing *r* and this reduces conductivity. Overall, κ from the three approaches qualitatively agree with each other up to the highest salt concentration. The quantitative comparison reveals two trends. There is a quantitative agreement between SOL and COM at all salt concentrations. In contrast, κ from CNE is 2–3 times lower than that from SOL and COM above *r* = 0.10. The reason for this is discussed below.

**Figure 5:**
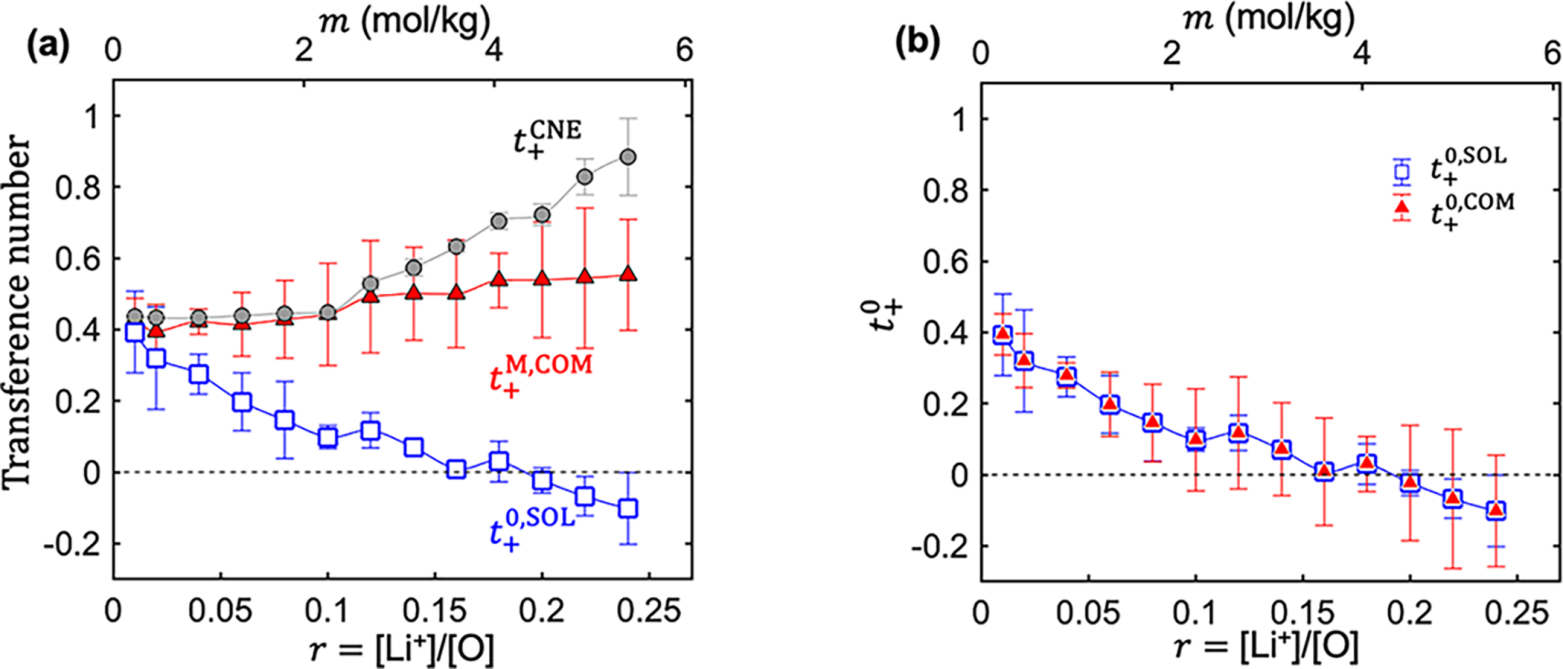
Cation transference numbers from the three approaches as a function of salt concentration *r*. (a) Transference numbers as defined in eq 4. (b) Transference numbers under the solvent reference frame obtained directly from SOL simulations, *t*_+_^0,SOL^, compared with *t*_+_^0,COM^, obtained by converting *t*_+_^M,COM^ to *t*_+_^0,COM^ using eq 7. While the values obtained by the SOL and COM approaches are indistinguishable, the error bars associated with the COM approach are smaller in most cases.

The transference numbers were calculated from the transport coefficients using eq 4, and the results are shown in Figure 5. *t*_+_^M,COM^ deviates from *t*_+_^0,SOL^ at almost all salt concentrations, 0.01 < *r* < 0.25. The deviation between them increases with increasing salt concentration. While *t*_+_^M,COM^ is nearly independent of salt concentration, *t*_+_^0,SOL^ decreases with increasing salt concentration and is negative above *r* = 0.20. *t*_+_^0^ obtained from simulations are generally consistent with the results measured by experiments.^80^ The effect of reference frames on transference numbers is discussed in refs (1) and (2), and the relationship between *t*_+_^0^ and *t*_+_^M^ is given by
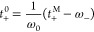
7where ω_*–*_ and ω_0_ are the mass fractions of anion and solvent in the system, respectively. Equation 7 enables calculating *t*_+_^0^ from COM simulations. *t*_+_^0^ is thus obtained as *t*_+_^0,COM^. In Figure 5b, we compare *t*_+_^0,COM^ and *t*_+_^0,SOL^ and find quantitative agreement at all salt concentrations. This agreement demonstrates the importance of recognizing the underlying reference frame for each computational scheme. The agreement between SOL and COM has also been observed in recent simulations of carbonate electrolytes^81^ and polymer electrolytes.^82^ The matrix that relates transport coefficients in SOL and COM approaches was derived in ref (82).

Next, we discuss *t*_+_^CNE^. There is no general relationship between the fixed point reference frame in the CNE approach and those defined in SOL and COM approaches. Returning to Figure 5a, we compare *t*_+_^CNE^ with *t*_+_^M,COM^ and *t*_+_^0,SOL^. *t*_+_^M,COM^ and *t*_+_^CNE^ agree quantitatively below *r* = 0.10, but significant deviations arise at higher concentrations. *t*_+_^0,SOL^ and *t*_+_^CNE^ deviate substantially at nearly all salt concentrations. The agreement between *t*_+_^M,COM^ and *t*_+_^CNE^ below *r* = 0.10 rests on two simplifications. (1) The electrolytes in this regime can be accurately modeled as a collection of uncorrelated clusters. (2) The center of mass of the simulated system is more-or-less fixed in space. The extent to which these simplifications can be applied to other electrolytes remains to be established. At high salt concentrations above *r* = 0.10, the CNE approach breaks down, indicating that electrolytes in this regime cannot be accurately modeled as a collection of uncorrelated clusters. This can be seen in both the conductivity (Figure 4) and transference number (Figure 5a).

**Figure 6:**
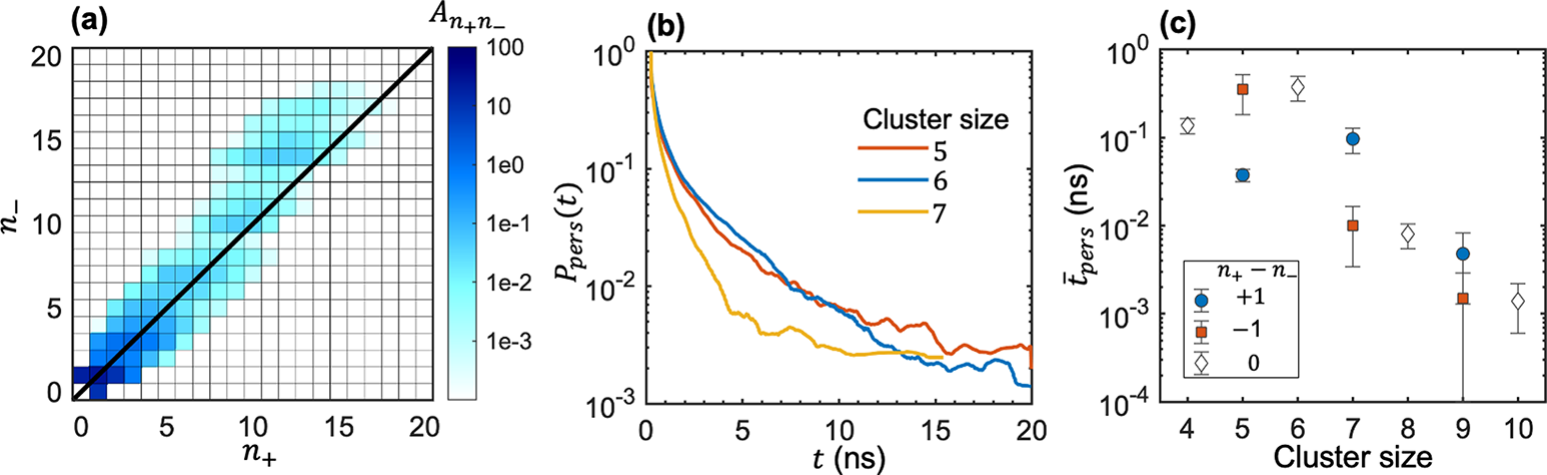
Ion clustering at high salt concentrations, *r* = 0.20. (a) Distribution of average number of ion clusters, *A*_*ij*_, made of *i* = *n*_+_ cations and *j* = *n*_–_ anions. (b) Probability distribution of the persistence time (*P*_*Pers*_) for clusters made of three anions (*n*_*–*_ = 3, *n*_+_ + *n*_*–*_ = 5, 6, 7). (c) Average persistence time (*t̅*_*Pers*_) as a function of cluster size for clusters near the diagonal line in (a), *n*_+_ – *n*_*–*_ = ± 1 or *n*_+_ – *n*_*–*_ = 0.

To reconcile the deviation between CNE and SOL/COM approaches at high salt concentrations, the clustering between ions was examined.^51,76^ For completeness, we discuss the results obtained at *r* = 0.20. Figure 6a shows the cluster distribution where the average number of clusters of a given type with *k* = *n*_+_ cations and *l* = *n*_*–*_ anions, *A*_*kl*_, is plotted as a function of *n*_*+*_ and *n*_–_. This distribution is biased toward negatively charged clusters, which was postulated to result in negative transference numbers.^11,76^ For practical reasons, the originators of the CNE approach tracked the motion of ion clusters whose average number is larger than 0.5 in the simulation system, i.e., *A*_*kl*_ > 0.5.^51^Figure 6a shows that several large clusters are omitted in such a computational scheme; all clusters with *n*_+_ + *n*_–_ > 7 are not included in the calculation scheme proposed in ref (51). We next examine the lifetimes of the existing clusters, which is quantified by the persistent time probability distribution. *P*_Pers_(*t*) is the lifetime distribution of all clusters with the targeted composition. An example of results thus obtained is presented in Figure 6b, where the persistence time distributions of some clusters made of three anions are shown. Very few of these clusters have a persistence time that meets the 20 ns time window required for computing *D*_*kl*_. The persistence time distribution analysis was repeated for several cluster sizes. Figure 6c shows the effect of cluster size on the average persistence time (*t̅*_*Pers*_ = ∫_0_^∞^*tP*_Pers_(*t*)d*t*) obtained for clusters along the diagonal line in Figure 6a with *n*_+_ – *n*_*–*_ = 0, and along off-diagonal lines with *n*_+_ – *n*_*–*_ = ± 1. *t̅*_Pers_ decreases almost exponentially with cluster size, indicating that the motion of larger clusters is increasingly challenging to track in the CNE approach.

As CNE only includes *D*_*ij*_^*c*^ from small clusters, the computed κ is thus several times lower than that from the two Onsager approaches at high salt concentrations. In addition, it can be observed in Figure 6a that small clusters are more positively charged in this electrolyte, e.g., *A*_21_ for *n*_+_ = 2 and *n*_*–*_ = 1 is larger than *n*_+_ = 1 and *n*_*–*_ = 2. This artificially biases the calculations of *t*_+_^0^ toward positive values. This gives a *t*_+_^CNE^ value of +0.88 at *r* = 0.24. The two Onsager-based approaches give *t*_+_^0^ = −0.10 at the same salt concentration.

## IV Conclusion and Outlook

We have critically examined three approaches for determining the cation transference number in electrolytes, *t*_+_. This is a transport property defined as the fraction of current carried by the cation under an applied field in an electrolyte of uniform concentration; the experimental approaches for measuring the transference number require out-of-equilibrium experiments under an applied field. In the simple case of a dissociated salt in a solvent, the transference number is defined in terms of the average velocities of the three species—the cation, the anion, and the solvent—and the cation current is proportional to the cation concentration and velocity. We consider two definitions of the *t*_+_: (1) *t*_+_^0^ based on the solvent velocity as the reference and (2) *t*_+_^M^ based on the mass average velocity as the reference. We discuss two approaches to obtain these transference numbers from equilibrium MD simulations:^47,52^ (1) SOL wherein the displacements of species are tracked using the center-of-mass of all solvent molecules in the simulation box as the reference and (2) COM wherein the displacements of species are tracked using the center-of-mass of all species in the simulation box as the reference. The central quantities used in these approaches are transport coefficients (*W*_*ij*_ and *F*_*ij*_) that represent the correlated motions of the species; ion-containing clusters that form and breakup are accounted for naturally in these transport coefficients. The ensemble averages within eqs 3a and 3b for *W*_*ij*_ and *F*_*ij*_ are collective properties of the entire system rather than those averaged over different particles such as MSD in self-diffusion coefficients. The two simulation approaches give the same transference number in the limit of infinite dilution, but they diverge significantly as the concentration of charged species in the electrolyte increases. However, the two approaches agree quantitatively at all concentrations for the case of LiTFSI/tetraglyme when the difference in reference frames is accounted for (eq 7). One expects to find such an agreement in all electrolytes.^2^

It is difficult to use intuition to interpret collective properties such as *W*_*ij*_ and *F*_*ij*_. If the charged species are fully dissociated and decoupled from each other, then transport coefficients can be obtained directly from self-diffusion coefficients of the ions using the Nernst–Einstein approach. The development of the cluster Nernst–Einstein (CNE) approach is significant because it provides insights into the nature of ion-containing clusters in concentrated electrolytes that cannot be modeled using the Nernst–Einstein approach. In the CNE approach, different kinds of clusters are identified and transport properties are calculated based on their self-diffusion coefficients. The CNE approach as proposed determines *t*_+_ relative to a static reference frame; the relationship between this frame and the internal reference frames used in the SOL and COM approaches appears to be nonuniversal and may depend on factors such as the molar mass of the species and the extent of interspecies coupling. For the LiTFSI/tetraglyme case, the CNE and COM approaches agree at low concentrations, up to *r* = 0.10. It is conceivable that increasing the simulation time of CNE may lead to agreement between the two approaches over a wider concentration window as this will enable capturing larger ion clusters that have much smaller chance to persist longer than the chosen time window. CNE, COM, and SOL approaches must agree with each other in the limit of infinite dilution.

The agreement between two Onsager approaches demonstrated in LiTFSI/tetraglyme electrolyte as well as that implied by recent work suggests they are robust methods for quantifying *t*_+_^0^ in electrolyte systems. The use of Onsager transport coefficients to understand transport behaviors such as cation transference in electrolyte systems is advocative from many aspects. Transport coefficients from Onsager approaches denote the species correlations at the atomistic level. Recent literature suggest these coefficients act as an important intermediate that bridges the understanding from molecular structures/interactions to macroscopic transport properties.^81,83^ The Onsager transport coefficients are also in parallel and compatible with the widely used Stefan–Maxwell diffusion coefficients in electrolyte characterization.^52^ The cluster Nernst–Einstein approach provides an intuition to approximate ion correlations from the motion of ionic clusters. However, the accuracy of quantifying *t*_+_^0^ at concentrated solutions highly depends on the cluster distributions as the types of clusters that can be effectively gathered is limited to small ones. We suggest that rigorous quantification of *t*_+_^0^ in molecular simulation should better be based on Onsager approaches.

The SOL and COM approaches are not limited to electrolytes consisting of a binary salt and a solvent. They can be applied to electrolytes with polymeric solvents, three or more kinds of ions, and two or more kinds of solvent molecules. As the number of species increases, one would have to quantify additional transport properties. We note in passing that the standard electrolyte used in lithium batteries contain two solvents (with numerous additives that are often trade secrets). For the two solvents case, the SOL reference frame seems appropriate for quantifying the transference numbers with respect to both solvents, as they can be directly obtained from simulations.^47^

All approaches for determining transport coefficients from simulations such as SOL, COM, and CNE will benefit from the development of more sophisticated machine learning-based algorithms.^46,84^

Quantitative agreement between the transference number obtained from simulation and that measured by experiment^51,52,57,80−82,85−88^ establishes the foundation for analyzing *t*_+_^0^ through simulation. While simulations alone can be used to quantify *t*_+_^0^ to screen new electrolytes, the variability of force fields and simulation methods does not guarantee that the modeled ion transport is an accurate representation of reality. Insights regarding species correlations from simulation must be corroborated by experiments such as scattering and spectroscopy.^89^ To our knowledge, however, comparisons between experiments and simulations are limited to values of transference numbers. Knowledge of species correlations will enable determination of the distribution of species velocities that underlie the averages presented in eq 1. Experimental determination of the heterogeneous motion of different transient clusters (see Figure 1) in concentrated electrolytes remains an important unmet challenge.
